# Relationship between the Manner of Mobile Phone Use and Depression, Anxiety, and Stress in University Students

**DOI:** 10.3390/ijerph15040697

**Published:** 2018-04-08

**Authors:** Aleksandar Višnjić, Vladica Veličković, Dušan Sokolović, Miodrag Stanković, Kristijan Mijatović, Miodrag Stojanović, Zoran Milošević, Olivera Radulović

**Affiliations:** 1 Department of Social Medicine, Faculty of Medicine, University of Niš, 18000 Niš, Serbia; vladica.velickovic@gmx.com (V.V.); nuna0203@gmail.com (O.R.); 2 Institute of Public Health of Niš, 18000 Niš, Serbia; drmstojanovic@gmail.com (M.S.); zormilzoran@gmail.com (Z.M.); 3 Department of Biochemistry, Faculty of Medicine, University of Niš, 18000 Niš, Serbia; soko@medfak.ni.ac.rs; 4 Department of Psychiatry and Medical Psychology, Faculty of Medicine, University of Niš, 18000 Niš, Serbia; adolescencija@gmail.com; 5 Clinic for Mental Health Protection, Clinical Centre of Niš, 18000 Niš, Serbia; 6 Department of Public Health, Faculty of Medicine, Catholic University of Sacred Heart, 00168 Rome, Italy; dr.kristijanm@gmail.com; 7 Department of Medical Statistics and Informatics, Medical Faculty of University, 18000 Niš, Serbia

**Keywords:** mobile phone, mental health, depression, anxiety, stress

## Abstract

*Objectives*: There is insufficient evidence regarding the potential risk of mobile phone use on mental health. Therefore, the aim of this research was to examine the relationship between mobile phone use and mental health by measuring the levels of depression, anxiety, and stress among university students in Serbia and Italy. *Methods*: This cross-sectional study was carried out at two distinguished universities in Serbia and Italy from March to May of the 2015/2016 academic year and included 785 students of both genders. The questionnaire was compiled and developed from different published sources regarding the manner and intensity of mobile phone use, along with the Depression Anxiety Stress Scale (DASS 42) for measuring psychological health. The statistical analysis of the data included the application of binary logistic regression and correlation tests. *Results:* Statistical analysis indicates that anxiety symptoms are somewhat more present in younger students (odds ratio (OR) = 0.86, 95% confidence interval (CI): 0.76–0.96), in those who send more text messages (SMSs) (OR = 1.15, 95% CI: 1.11–1.31), and in those who browse the internet less frequently (OR = 0.84, 95% CI: 0.73–0.95). Stress is more common in students who make fewer calls a day (OR = 0.79, 95% CI: 0.64–0.97), as well in those who spend more time talking on the mobile phone per day (OR = 1.28, 95% CI: 1.12–1.56). The strongest predictor of high stress levels was keeping the mobile phone less than 1 m away during sleeping (OR = 1.48, 95% CI: 1.12–2.08). *Conclusions:* The results indicated that the intensity and modality of mobile phone use could be a factor that can influence causal pathways leading to mental health problems in the university student population.

## 1. Introduction

Due to their wide-spread availability, mobile phones have become a necessity (something “without which you cannot”). But recent studies have shown that the use of mobile phones is associated with headaches, neurovegetative dystonia, irritability, sleep disorders, fatigue, and dizziness [1,2,3,4,5]. The nervous system has been proven to be the most sensitive to the effects of the electromagnetic field of mobile phones [6,7]. Certain harmful effects of the mobile phone signal during electroencephalogram (EEG) testing have also been observed, but these findings are still under discussion [8,9,10,11,12,13].

Some previous studies have also shown high rates of mental disorders (typically depression, anxiety, and stress) among university students around the world [14,15], who are enduring a special period of great challenges and risks.

In modern society, there is a constant concern that the ever-increasing use of mobile phones may have harmful effects on some health aspects; on the other hand, there are numerous positive/useful examples of mobile phone use (mostly smart phones) in medicine, education, and other fields [12,16].

The results of a rapid literature search in MEDLINE and MEDLINE in progress suggest that there is a small number of primary research studies regarding the potential risk of mobile phone use on mental health, and consequently, there is insufficient foundation of evidence in this area. Therefore, this study takes one step towards shedding light on this complex phenomenon.

The aim of this research was to explore the manner and intensity of the use of mobile phones and examine its effects—especially the effects of long-term mobile phone use—on certain mental health aspects of university students in Serbia and Italy, by measuring levels of depression, anxiety, and stress.

## 2. Methods

### 2.1. Participants

The cross-sectional study was carried out at two distinguished universities in two countries—the University of Niš, Faculty of Medicine, in Serbia and the Catholic University of the Sacred Heart, Faculty of Medicine and Surgery in Rome, Italy—from March to May of the 2015/2016 academic year. All of the participants were assessed by using a questionnaire, and the total number of responding participants was 827, of whom 42 were rejected due to incomplete responses. Thus, the study ultimately included 785 randomly selected university students (253 males and 532 females).

### 2.2. Procedure

The students reported their socioeconomic characteristics, lifestyle habits, attitudes, and health assessment using self-administered questionnaires, which also provided answers to questions about the manner, purpose, and intensity of mobile phone use.

Symptoms of depression, anxiety, and stress were assessed using the Depression Anxiety Stress Scale (DASS 42), a device for measuring psychological health [17,18].

The survey was performed in classrooms by trained assistants (interviewers) and was intended to last a maximum of 20 min, including the time needed for instructions.

### 2.3. Measures

The DASS device is a set of three self-reporting scales designed to measure the negative emotional states of depression, anxiety, and stress. Each of the three scales contains 14 items, divided into the subscales of 2–5 items with similar content. DASS is designed to be used for further defining, understanding, and measuring all present and clinically significant emotional states in the examinees [17,18].

The students were asked to mark from 0 (none) to 3 (mostly or almost always) the extent to which they have experienced each of the listed conditions during the previous week. The score results of depression, anxiety, and stress were calculated by adding the points for each relevant scale. The result was then calculated for every student and for each of the subscales, according to the score matrix, and then evaluated as per the severity-rating index below (Table 1).

The reliability scores of the scales in terms of Cronbach’s alpha scores rate the depression scale at 0.91, the anxiety scale at 0.84, and the stress scale at 0.90 in the normative sample. The means for each scale are 6.34 (SD 6.97) for depression, 4.7 (SD 4.91) for anxiety, and 10.11 (SD 7.91) for stress.

The frequency of calls (conversations) per day (How many calls on average do you make and receive a day with your mobile phone?) was assessed by using the following options: 0–5 calls, 6–10 calls, 11–20 calls, 21–30 calls, and >30 calls. The frequency of SMSs per day (How many text messages do you send or receive on your mobile phone?) was assessed by using the following options: 0–10 text messages (SMSs), 11–20 SMSs, 21–50 SMSs, 51–100 SMSs, and >100 SMSs.

The subjects also reported their time spent on the internet, time spent using various applications, and time spent playing games (If you use other mobile phone functions, how much time do you spend using those functions?) by using the options: no, several times a week or less, <1 h a day, 1–2 h a day, and >2 h a day. The distance of the mobile phone during sleep (Is your mobile phone less than 1 m away while you sleep?) was assessed with yes or no answers, and the total time spent on the phone (How much time on average do you use a mobile phone?) was assessed by using the options: <30 min, 30 min–1 h, 1–2 h, 2–3 h, and >3 h.

### 2.4. Statistical Analysis

All of the data were entered into Excel spreadsheets (Microsoft Office 2003, Microsoft, Redmond, DC, USA) by several teams each consisting of two people, whereby cross-checking was done for every given survey. The statistical analysis was performed using the SPSS 17.0 program (SPSS Inc., Chicago, IL, USA) in Windows 7 Ultimate. The research results were presented in tables.

The statistical analysis of the data included the application of descriptive tests and analytical parametric tests, as well as binary logistic regression tests and correlation tests. The descriptive statistics were performed to report the analysis of the data that were presented as mean and standard deviations. The categorical variables were shown as frequency and percentages. The independent *t*-test was used to compare the parametric variables between the genders. Pearson and Spearman correlations were used to determine the strength of the relationships between the examined variables. Binary logistic regression was used to estimate the odds ratios (ORs) and 95% confidence intervals (CIs) of the independent and interactive relationships of several prediction variables with depression, anxiety, and stress. The statistical significance was set at *p* < 0.05.

### 2.5. Ethics

The study procedures were carried out in accordance with the Declaration of Helsinki. The Ethical Committee of the Faculty of Medicine of the University of Niš (14-5785-3) approved the study. All subjects were informed about the study, and all provided informed consent.

## 3. Results

### 3.1. General Characteristics of Participants and an Overview of the Manner and the Intensity of Mobile Phone Use

The study included 785 participants, 253 male (32.2%) and 532 female (67.8%) students. The mean value of the year of study was 2.25 (SD 1.45), while the mean age was 20.75 (SD 2.60). The *t*-tests did not show any significant differences between students of both genders in either of these two values. There were 600 students from Serbia, out of whom 212 (35.3%) were male and 388 (64.7%) were female, and there were also 185 students from Italy, out of whom 41 were male (22.2%) and 144 were female (77.8%).

Out of the total number, 572 students (72.9%) had only one mobile phone. Moreover, 259 of them (33.0%) stated that they felt certain harmful effects of longer mobile phone use. Headphones were not used by 561 (71.5%) students, whereas Bluetooth was used by 38 (4.8%) and wire headphones were used by 186 (23.7%) students. Health issues were reported by 67 (8.5%) students. In addition, 657 students (83.7%) believed that excessive use of mobile phones causes health problems. The mobile phone was kept at a distance of less than 1 m during sleep by 410 (52.2%) students. Some other characteristics of the surveyed students are shown in Table 2.

In addition to talking and sending/receiving SMS messages, mobile phones were most frequently used by students (for more than 1 h a day) for internet browsing (43.7%), for listening to music (36.2%), for some applications (24.0%), and for playing games (12.8%).

### 3.2. Correlation between Certain Characteristics of the Surveyed Students and Some Habits Related to Mobile Phone Use

The associations between some characteristics of the surveyed students and their habits related to the use of mobile phones were examined by a Pearson correlation coefficient (i.e., by Spearman’s rho rank correlation coefficient). Preliminary analyses were conducted in order to check the assumptions of normality, linearity, and homogeneity of the variants. The following correlations were calculated (Table 3).

The higher amount of weekly allowance is correlated with more frequent calls, more time spent using mobile phones daily, a greater number of SMSs, and more frequent use of the internet, apps, and games. Those students who go to sleep later make more phone calls during the day, while physically more active students exchange fewer messages. Also, later sleep time is correlated with more internet browsing and longer hours of sleep with more games played (Table 3).

### 3.3. Prediction of the Levels of Depression, Anxiety, and Stress Based on the Parameters Related to the Manners and Intensity in Which Mobile Phones Are Used

Moderate, severe, or extremely severe levels of depression symptoms were reported by 166 students (21.2%) (Table 4). “Above mild” anxiety symptoms were reported by 287 (26.6%) students, while 207 (26.3%) students were exposed to moderate, severe, or extremely severe stress.

Direct logistic regression was conducted in order to estimate the effects of several factors on the probability that the students would respond positively to questions about depression, anxiety, or stress. First, in SPSS we performed “transform variable” into two answers by classifying the scores on the DASS 42 in two categories, whereby answers “normal” and “mild” were classified as NO, and the remaining three (moderate, severe, and extremely severe) were classified as YES.

The model (a group of prediction variables—how well it predicts or explains the “outcome”) contains 10 independent variables: gender, country, year of study, number of calls per day, total time spent on the phone per day, number of messages per day, distance of mobile phone during sleep, time spent on the internet, time spent using apps, and time spent playing games. The whole model (along with all the predictors) was statistically significant, namely: for depression: χ^2^ (10, *N* = 785) = 42.21, *p* < 0.005; for anxiety: χ^2^ (10, *N* = 785) = 51.70, *p* < 0.005; and for stress: χ^2^ (10, *N* = 785) = 58.92, *p <* 0.005.

This indicates that the model distinguishes those respondents who are from those who are not sorted, so that they have some of the symptoms.

The model explains between 6.8% (r^2^ Cox and Snell) and 10.6% (r^2^ Nagelkerke) variance in the existence of depression symptoms, between 12.6% (r^2^ Cox and Snell) and 17.2% (r^2^ Nagelkerke) variance in the existence of anxiety symptoms, and finally between 12.3% (r^2^ Cox and Snell) and 17.9% (r^2^ Nagelkerke) variance in the existence of stress. The assumptions of collinearity and singularity were satisfied, and non-typical points were also checked.

As Table 5 shows, eight independent variables provided a unique statistically significant contribution to some of three presented models (gender, year of study, number of calls per day, time spent on the phone per day, number of SMSs per day, distance of mobile phone during sleep, browsing the internet, and playing games).

The strongest predictor of whether a student has high levels of depression symptoms was gender, where the odds ratio was OR = 1.57. This shows that male students responded that they have symptoms of depression 1.57 times more often, with all other factors in the model being equal. Also, it has been observed that depression symptoms are more common in those students who make fewer calls during the day (OR = 0.7), send more SMSs (OR = 1.31), and browse the internet less frequently on the mobile phone (OR = 0.85).

Anxiety symptoms are somewhat more present in younger students (OR = 0.86), in those who send more SMSs (OR = 1.15), browse the internet less frequently (OR = 0.84), and play games on their mobile phones less frequently (OR = 0.85).

Stress is more common in students who make fewer calls a day (OR = 0.79), as well as in those who spend more time talking on the mobile phone per day (OR = 1.28), younger students (OR = 0.8), and those who play games less frequently (OR = 0.83). The strongest predictor of high stress levels was keeping the mobile phone less than 1 m away during sleeping, where odds ratio was OR = 1.48. This shows that students who keep their mobile phones less than 1 m away while they slept reply 1.48 times more often that they are exposed to stress, with all other factors in the model being equal. The country of origin (Italy and Serbia) played no part in any of the cases.

## 4. Discussion

The study findings suggest that a stronger predictor of depression among students in both countries is male gender. Those results are not in line with the studies in the general population, even with some prospective studies, in which female gender was the risk factor for depression [19,20]. In addition, we found a pattern that a depressed male student makes fewer calls and browses the internet less frequently on the mobile phone and sends more SMSs. In general, our findings confirm the results obtained by several studies reporting on the relationship between mobile phone use and depression symptoms [21,22,23,24,25].

However, it is important to emphasize that some studies indicate that it is addiction to mobile phones in college-age students that leads to depression and anxiety, not the use of mobiles [21,25]. The main question of the attributable risk fraction coming from the mobile phone devices “per se”, presumably because of electromagnetic radiation, still needs to be addressed. Isolating such effect from the multivariate background of underlying causes governing depression, which are probably mediated by several levels of hierarchical and time-dependent factors, is a challenging task, even for prospective studies.

Furthermore, we found similar patterns in mobile phone usage among students with anxiety symptoms. Namely, those students also send many more SMSs and spend less time browsing the internet, and in this case, they are students of younger age. However, those patterns in both depressed students and students with anxiety symptoms can be triggered by smaller monthly economic budgets rather than by the use of mobile phones itself. Some explanatory studies found that students with higher anxiety are more likely to use mobile phones as a compensatory attachment target [26]. Therefore, the correlation between anxiety and mobile phone use from our study is still an important question for future prospective studies as well.

Those patterns characteristic of depression and anxiety are not associated with stress levels, for which the most significant predictor is the physical distance of the phone from bed during sleep (less than 1 m). Those findings are in line with similar findings from other research [23]. Current recommendations suggest that the cell phone should be at least three feet away from the body during sleep [27].

Several studies used EEG measurements to estimate more precisely the impact of mobile phones on sleep quality [13,28,29,30,31]. The results are currently rather inconclusive or inconsistent, and one study even found that sleep quality improved due to mobile phones [28].

A large German research study from 2006 suggested that the worst sleep quality can be found among a particular type of study participants, and those findings cannot be generalized for the overall population [32]. Despite the reported results, several studies found an alteration in the circadian system and even changes in cardiac rhythms caused by mobile phones during sleeping hours [33].

Whether there is a predication for such alterations or those effects are typical for the overall population is an open question. The pathogenesis of the described conditions is still not completely understood, but current studies underline their causal interdependence [34].

Different kinds of stress are risk factors for developing an anxiety disorder, and these can, in turn, cause or worsen sleep disorders. Similarly, stress can trigger depression, which can then be complicated by anxiety. Several animal and human basic research studies indicate the effects of using mobile phones on cognition and brain functions [35,36,37].

Our and similar research confirm the findings on the manifestation level of those complex conditions. Even if the exact underlying mechanisms are not clear because the conditions modified by mobile phones per se still do not have a fully explained etiopathogenesis, the quantity of new evidence indicates that mobile phone usage is part of multiple underlying causal links to those described conditions.

Keeping in mind that different forms of mobile communication reshape our way of living and the culture of communication, these and similar findings should alert public health policy makers to adequately promote and stimulate more in-depth and comprehensive research in the area. Although “nomophobia” does not appear in the current Diagnostic and Statistical Manual of Mental Disorders, Fifth Edition (DSM-5) [38], it has been proposed as a new concept to describe a specific phobia of being without mobile phone contact. This condition has very limited treatment options and can cause serious social, psychological, and general health problems in considerably significant populations [39]. Current treatments include cognitive-behavioral psychotherapy combined with pharmacological interventions, and in the future, those kinds of conditions can have a significant budget impact on health care systems around the world. The main question raised after this research is the need to distinguish more accurately two potential causes of those mental problems. Namely, those conditions can be caused, modified, or affected by electromagnetic radiation, or they can be more high-level, new age, compensatory attachment target conditions developed independently of electromagnetic radiation. As a third possibility, both mechanisms could contribute to the development and modification of described mental health problems.

In evaluating the results, several limitations of the study should be considered. The cross-sectional design of the survey does not allow appropriate insight into the causal relationships between the factors included in the analysis. The validation of the results of this study requires a longitudinal study. Furthermore, the results presented in this study cannot be generalized to the total population, considering that the existing patterns in university student populations can differ from the general population. The self-reporting bias, as a common problem in observational research studies, was present in this study as well due to the use of self-reporting scales [24]. Due to the nature of questionnaire-based research, recall bias is certainly present in the study [40]. In addition, we did not take into consideration differences in relation to various cell phone brands, whereas some studies could find this kind of relationship. A distinction between smart phones and regular cell phones was not considered as well, and even this can be an issue of importance.

On the other hand, the limited, published literature regarding the research questions addressed in this study and the similar responses obtained from two different countries (Italy and Serbia) may serve as a solid foundation for future research.

## 5. Conclusions

The presented findings strongly indicate that the intensity and the modality of mobile phone use could be a factor that can influence causal pathways leading to mental health problems in university student populations. The general trend of increased use of mobile phones, especially in university students and young adolescents therefore dictates the need for more comprehensive longitudinal research. However, even on this level of investigation, it is more than evident that a certain degree of caution must be considered among high-frequency users.

## Figures and Tables

**Table 1:** Depression Anxiety Stress Scale (DASS 42) score.

Level	Depression	Anxiety	Stress
Normal	0–9	0–7	0–14
Mild	10–13	8–9	15–18
Moderate	14–20	10–14	19–25
Severe	21–27	15–19	26–33
Extremely severe	28+	20+	34+

**Table 2:** General characteristics of the surveyed students.

Place of Living	With Parents	In Students’ Dormitory	Alone in an Apartment	With Roommate(s) in an Apartment	
	*n* = 305 (38.9%)	*n* = 56 (7.1%)	*n* = 175 (22.3%)	*n* = 249 (31.7%)	
Physical activity	Almost every day	2–3 times a week	2–4 times a month	Almost never	
	*n* = 102 (13.0%)	*n* = 191 (24.3%)	*n* = 244 (31.1%)	*n* = 248 (31.6%)	
Sleep time	by 22 h	by 24 h	by 02 h or later		
	*n* = 32 (4.1%)	*n* = 368 (46.9%)	*n* = 385 (49.0%)		
Amount of sleep	up to 5 h	6–8 h	9 and more hours		
	*n* = 117 (14.9%)	*n* = 607 (77.3%)	*n* = 61 (7.8%)		
Calls per day	0–5 calls	6–10 calls	11–20 calls	21–30 calls	>30 calls
	*n* = 255 (32.5%)	*n* = 310 (39.5%)	*n* = 147 (18.7%)	*n* = 42 (5.4%)	*n* = 31 (3.9%)
Daily mobile phone use	Less than 30 min	30 min–1 h	1–2 h	2–3 h	More than 3 h
	*n* = 257 (32.7%)	*n* = 298 (38.0%)	*n* = 138 (17.6%)	*n* = 57 (7.3%)	*n* = 35 (4.5%)
SMSs per day	0–10 SMS	11–20 SMS	21–50 SMS	51–100 SMS	More than 100
	*n* = 198 (25.2%)	*n* = 239 (30.4%)	*n* = 191 (24.3%)	*n* = 97 (12.4%)	*n* = 60 (7.6%)
Position of phone when not used	Pocket	Bag or phone case	Close to oneself	Away from oneself	
	*n* = 174 (22.2%)	*n* = 192 (24.4%)	*n* = 92 (11.7%)	*n* = 327 (41.7%)	

**Table 3:** Correlations between certain characteristics and cell phone use related habits.

Variables	Calls per Day	Conversations per Day	SMS per Day	Time on the Internet	Time Using Apps	Time Playing Games
Age	0.140 **	0.047	−0.130 **	−0.028	−0.030	0.089 *
*p*	0.000	0.185	0.000	0.434	0.394	0.013
Faculty department	0.072 *	0.018	0.142 **	0.102 **	0.006	0.014
*p*	0.042	0.608	0.000	0.004	0.865	0.697
Place of living	−0.033	0.062	0.069	−0.038	−0.094 **	−0.078 *
*p*	0.353	0.084	0.053	0.289	0.008	0.028
Weekly budget	0.226 **	0.184 **	0.091 *	0.083 *	0.082 *	0.078 *
*p*	0.000	0.000	0.012	0.021	0.023	0.030
Physical activity	0.022	−0.041	−0.083 *	0.008	−0.010	0.050
*p*	0.543	0.251	0.020	0.813	0.777	0.158
Sleep time	0.084 *	0.047	0.053	0.090 *	0.088 *	−0.019
*p*	0.019	0.187	0.136	0.011	0.014	0.595
Amount of sleep	0.049	0.013	0.068	−0.016	0.042	0.114 **
*p*	0.167	0.720	0.058	0.650	0.238	0.001
Number of mobile phones	0.114 **	0.128 **	0.053	−0.024	−0.015	0.051
*p*	0.001	0.000	0.140	0.497	0.680	0.157
First mobile phone	−0.104 **	−0.107 **	−0.159 **	−0.091 *	−0.165 **	−0.049
*p*	0.003	0.003	0.000	0.010	0.000	0.173
Position of phone when not used	−0.003	0.082 *	0.014	0.007	−0.021	−0.035
*p*	0.940	0.021	0.699	0.854	0.559	0.330

Notes: ** Correlation is significant at the 0.01 level, and * at the 0.05 level age, weekly budget, number of mobile phones, and first mobile phone are linear variables; faculty department (Medicine, Dentistry, Pharmacy); place of living (with parents, in students’ dormitory, alone in a rented or my own apartment, with someone else in a rented apartment); physical activity (almost every day, 2–3 times a week, 2–4 times a month, almost never); sleep time (until 22 h, until midnight, until 2 h or later); amount of sleep (up to 5 h, 6–8 h, 9 and more hours); position of phone when not used (pocket, bag or phone case, close enough to oneself, away from oneself).

**Table 4:** The scores of depression, anxiety, and stress with respect to students’ gender.

Symptom Levels	Depression			Anxiety			Stress		
	Total	Male	Female	Total	Male	Female	Total	Male	Female
		*n* %	*n* %		*n* %	*n* %		*n* %	*n* %
Normal	*n* = 521	166	355	*n* = 427	148	279	*n* = 471	171	300
	66.6%	65.6	66.7	54.4%	58.5	52.4	60.0%	67.6	56.4
Mild	*n* = 98	26	72	*n* = 71	23	48	*n* = 107	27	80
	12.5%	10.3	13.5	9.0%	9.1	9.0	13.6%	10.7	15.0
Moderate	*n* = 101	32	69	*n* = 148	34	114	*n* = 111	25	86
	12.9%	12.6	13.0	18.9%	13.4	21.4	14.1%	9.9	16.2
Severe	*n* = 40	18	22	*n* = 55	18	37	*n* = 74	27	47
	5.1%	7.1	4.1	7.0%	7.1	7.0	9.4%	10.7	8.8
Extremely severe	*n* = 25	11	14	*n* = 84	30	54	*n* = 22	3	19
	3.2%	4.3	2.6	10.7%	11.9	10.2	2.8%	1.2	3.6

**Table 5:** Prediction of the levels of depression, anxiety, and stress in the surveyed students.

Independent Variables	B	df	*p*	OR	95% CI for OR	
					Lower	Upper
Depression	Hosmer-Lemeshow test of goodness-of-fit (*p* = 0.479, for χ^2^ = 7.549, df = 8)					
Gender (1)	0.453	1	0.032	1.573	1.040	2.377
Country (1)	−0.464	1	0.058	0.629	0.393	1.015
Year of study	−0.006	1	0.920	0.994	0.875	1.129
Calls per day	−0.359	1	0.003	0.698	0.550	0.886
Time spent on the phone	0.002	1	0.982	1.002	0.808	1.244
SMS per day	0.273	1	0.001	1.314	1.124	1.536
Distance of cell during sleep (1)	0.135	1	0.465	1.145	0.796	1.646
Browsing	−0.163	1	0.038	0.849	0.728	0.991
Apps	0.156	1	0.077	1.168	0.983	1.389
Games	−0.113	1	0.193	0.893	0.753	1.059
Constant	−2.184	1	0.000	0.113	Correctly classified 78.5%	
Anxiety	Hosmer-Lemeshow test of goodness-of-fit (*p* = 0.267, for χ^2^ = 9.965, df = 8)					
Gender (1)	0.031	1	0.866	1.032	0.716	1.486
Country (1)	−0.231	1	0.243	0.793	0.538	1.170
Year of study	−0.154	1	0.009	0.857	0.764	0.962
Calls per day	−0.106	1	0.296	0.900	0.738	1.097
Time spent on the phone	0.105	1	0.264	1.111	0.924	1.337
SMS per day	0.140	1	0.040	1.151	1.107	1.313
Distance of cell during sleep (1)	0.033	1	0.837	1.034	0.752	1.421
Browsing	−0.179	1	0.008	0.836	0.733	0.953
Apps	0.110	1	0.164	1.116	0.956	1.302
Games	−0.236	1	0.002	0.789	0.678	0.919
Constant	−1.719	1	0.002	0.179	Correctly classified 67.6%	
Stress	Hosmer-Lemeshow test of goodness-of-fit (*p* = 0.339, for χ^2^ = 9.034, df = 8)					
Gender (1)	−0.028	1	0.893	0.972	0.646	1.464
Country (1)	−0.401	1	0.068	0.670	0.435	1.030
Year of study	−0.228	1	0.001	0.796	0.698	0.907
Calls per day	−0.241	1	0.027	0.786	0.635	0.973
Time spent on the phone	0.249	1	0.012	1.283	1.125	1.560
SMS per day	0.012	1	0.879	1.012	0.868	1.179
Distance of cell during sleep (1)	0.390	1	0.025	1.477	1.155	2.076
Browsing	−0.061	1	0.402	0.941	0.817	1.084
Apps	0.013	1	0.878	1.013	0.857	1.198
Games	−0.183	1	0.032	0.833	0.705	0.985
Constant	−2.916	1	0.000	0.054	Correctly classified 75.8%	

Notes: B—coefficient for the constant (“intercept”) in the null model; OR—odds ratio; CI—Confidence interval.
